# Latency Reversal 2.0: Giving the Immune System a Seat at the Table

**DOI:** 10.1007/s11904-020-00540-z

**Published:** 2021-01-12

**Authors:** Vidisha Singh, Amir Dashti, Maud Mavigner, Ann Chahroudi

**Affiliations:** 1 Department of Pediatrics, Emory University School of Medicine, Atlanta, GA USA; 2 Center for Childhood Infections and Vaccines of Children’s Healthcare of Atlanta and Emory University, Atlanta, GA USA; 3 Yerkes National Primate Research Center, Emory University Atlanta, Atlanta, GA USA

**Keywords:** HIV-1 cure, Immune-based therapeutics, Shock and kill, Latency reversal agents, SMAC, CD8 depletion

## Abstract

**Purpose of Review:**

For most people living with HIV (PLWH), treatment with effective antiretroviral therapy (ART) results in suppression of viremia below the limit of detection of clinical assays, immune reconstitution, reduced immune activation, avoidance of opportunistic infections, and progression to AIDS. However, ART alone is not curative, and HIV persists in a non-replicating, latent form. In this review, we provide a historical perspective on non-specific latency reversal approaches (LRA 1.0) and summarize recent advances in latency reversal strategies that target specific signaling pathways within CD4+ T cells or other immune cells to induce expression of latent HIV (immune-based latency reversal, or LRA 2.0).

**Recent Findings:**

The HIV reservoir is primarily composed of latently infected CD4+ T cells carrying integrated, replication-competent provirus that can give rise to rebound viremia if ART is stopped. Myeloid lineage cells also contribute to HIV latency in certain tissues; we focus here on CD4+ T cells as a sufficient body of evidence regarding latency reversal in myeloid cells is lacking. The immunomodulatory LRA 2.0 approaches we describe include pattern recognition receptor agonists, immune checkpoint inhibitors, non-canonical NF-kB stimulation, and transient CD8+ lymphocyte depletion, along with promising combination strategies. We highlight recent studies demonstrating robust latency reversal in nonhuman primate models.

**Summary:**

While significant strides have been made in terms of virus reactivation from latency, initial hopes for latency reversal alone to result in a reduction of infected cells, through viral cytopathic effect or an unboosted immune system, have not been realized and it seems clear that even effective latency reversal strategies will need to be paired with an approach that facilitates immune recognition and clearance of cells containing reactivated virus.

## Brief Historical Perspective on LRA 1.0

Following the initial descriptions of HIV persistence despite prolonged suppression of viremia in individuals on suppressive ART, approaches to target the latent reservoir by activating T cells were proposed. Clinical trials in the late 1990s–early 2000s used IL-2 alone or in combination with anti-CD3 antibodies to reverse HIV latency. This approach of global T cell activation had to be halted due to severe toxicity leading notably to acute renal failure, seizures, and hypothyroidism and was followed by more targeted strategies aimed at inducing HIV gene expression [1–4].

The next latency reversal agents (LRA) developed were designed to target HIV epigenetic silencing, a major regulator of viral latency that includes DNA methylation and histone post-translational modifications such as histone acetylation. The latency reversal activity of several histone deacetylase inhibitors (HDACi) including romidepsin, panobinostat, vorinostat, and valproic acid have been tested in clinical trials. Despite initial hopes, clinical studies with valproic acid did not demonstrate an impact on viral transcription [5, 6]. However, in 2012, it was reported that a single dose of vorinostat administered to 7 participants on suppressive ART induced a significant increase in cell-associated unspliced (CA US) HIV-1 RNA in resting CD4^+^ T cells [7]. Similar results were observed in follow-up studies during vorinostat multiple-dose therapy [8, 9]. It has to be noted that an increase in viremia, which is arguably the best read out for latency reversal (and to which we henceforth refer to as “on-ART viremia”; see Table 1 for assays used to quantify latency reversal), was not observed in any of these studies. In this regard, panobinostat treatment resulted in a qualitative increase in on-ART viremia compared to baseline in a subset of study participants [24]. Romidepsin infusions in 6 individuals on long-term ART also led to transient increases in plasma viral loads and CA US HIV-1 RNA levels in total CD4^+^ T cells [25]. The size of the viral reservoir was unchanged in each of these HDACi trials. Other epigenetic modifiers more recently explored as potential LRA include DNA and histone methyltransferase inhibitors (HMTi) targeting transcription initiation similarly to HDACi, as well as bromodomain and extra terminal (BET) inhibitors that promote transcription elongation. Notably, the HMTi’s chaetocin and BIX-01294 have been shown to increase HIV-1 recovery from ex vivo cultures of resting CD4^+^ T cells and small-molecule inhibitor of BET bromodomains such as JQ1, RVX-208, and PFI-1 appears to activate HIV transcription in latently infected Jurkat T cells [26–28]. These approaches have not yet been tested in PLWH. Collectively, the clinical studies to date show that LRA 1.0 approaches resulted in modest increases in CA US HIV-1 RNA, rare and limited increases in viremia, and no reduction of the viral reservoir in study participants maintained on suppressive ART.

**Table 1:** Assays evaluating LRA efficacy

Assay	Assay description	Advantages	Limirevions	References
Standard plasma viral load	Measures HIV/SIV RNA levels in plasma	▪ Rapid ▪ Relatively inexpensive ▪ Sensitive: LOQ of 20–60 copies of viral RNA/ml of plasma	▪ No indication of the origin of viral reactivation	[10–12]
Ultrasensitive plasma viral load	Measures HIV/SIV RNA levels in plasma	▪ Rapid ▪ Relatively inexpensive ▪ Ultrasensitive: LOQ down to 1 copy of viral RNA/ml of plasma	▪ No indication of the origin of viral reactivation	
Cell-associated HIV/SIV RNA qPCR or ddPCR	Measures HIV/SIV RNA levels in various cells. Multiple viral transcripts can be quantified: *-* Unspliced transcripts for specific genes - Chimeric host-virus readthrough transcripts (not transcribed from viral promoter) - TAR containing viral transcripts (transcriptional initiation) - Long LTR transcripts (transcriptional elongation) - Polyadenylated unspliced transcripts (completion of transcription) - Multiply spliced Tat-Rev transcripts (transcriptional initiation, elongation, and nuclear export; used as a surrogate of productive infection)	▪ Rapid ▪ Relatively inexpensive ▪ Sensitive ▪ Low cell input ▪ May demonstrate viral reactivation in specific cells	▪ Non-specific for replication-competent proviruses ▪ Can be performed with or without short ex vivo stimulation	[7, 13–16]
Tat/Rev Induced Limiting Dilution Assay (TILDA)	Measures the frequency of cells producing HIV/SIV multiply spliced Tat/Rev mRNA upon maximal stimulation ex vivo	▪ Sensitive ▪ Medium cell input	▪ Requires short ex vivo stimulation ▪ Non-specific for replication-competent proviruses ▪ More time-consuming than PCR ▪ More expensive than PCR	[17–19]
In situ RNA hybridization (RNAscope)	Measures HIV/SIV RNA levels in cells in situ	▪ Sensitive ▪ May demonstrate viral reactivation in specific cells and anatomical locations within tissues ▪ Low tissue input	▪ Non-specific for replication-competent proviruses ▪ More time-consuming than PCR ▪ More expensive than PCR assays	[20, 21]
RNA fluorescence in situ hybridization-flow cytometry (FISH-flow) assay	Measures intracellular HIV/SIV RNA at the single-cell level following activation	▪ Medium cell input ▪ Phenotypic characterization of individual cells	▪ Requires short ex vivo stimulation ▪ Non-specific for replication-competent proviruses ▪ More time-consuming than PCR ▪ More expensive than PCR assays	[22, 23]

Numerous assays have been developed to measure persistent and inducible HIV/SIV. In Table 1, we list the main assays used to evaluate the effectiveness of LRA in reactivating latent HIV/SIV. Due to their low cost and limited labor requirement, the two most commonly used assays to investigate LRA efficacy in reactivating HIV/SIV in pre-clinical and clinical studies are viral RNA in plasma and cell-associated viral RNA quantification by PCR. A panel of PCRs targeting various viral sequence regions can be used to distinguish different transcripts and blocks in transcription. In situ hybridization methods such as FISH-flow or RNAscope allow for additional phenotypic characterization of the cells producing viral RNA

*LOQ*, limit of quantitation; *qPCR*, quantitative polymerase chain reaction; *ddPCR*, droplet digital polymerase chain reaction; *TAR*, trans-activation response; *LTR*, long terminal repeat

## Immunomodulatory LRA 2.0

**Fig. 1:**
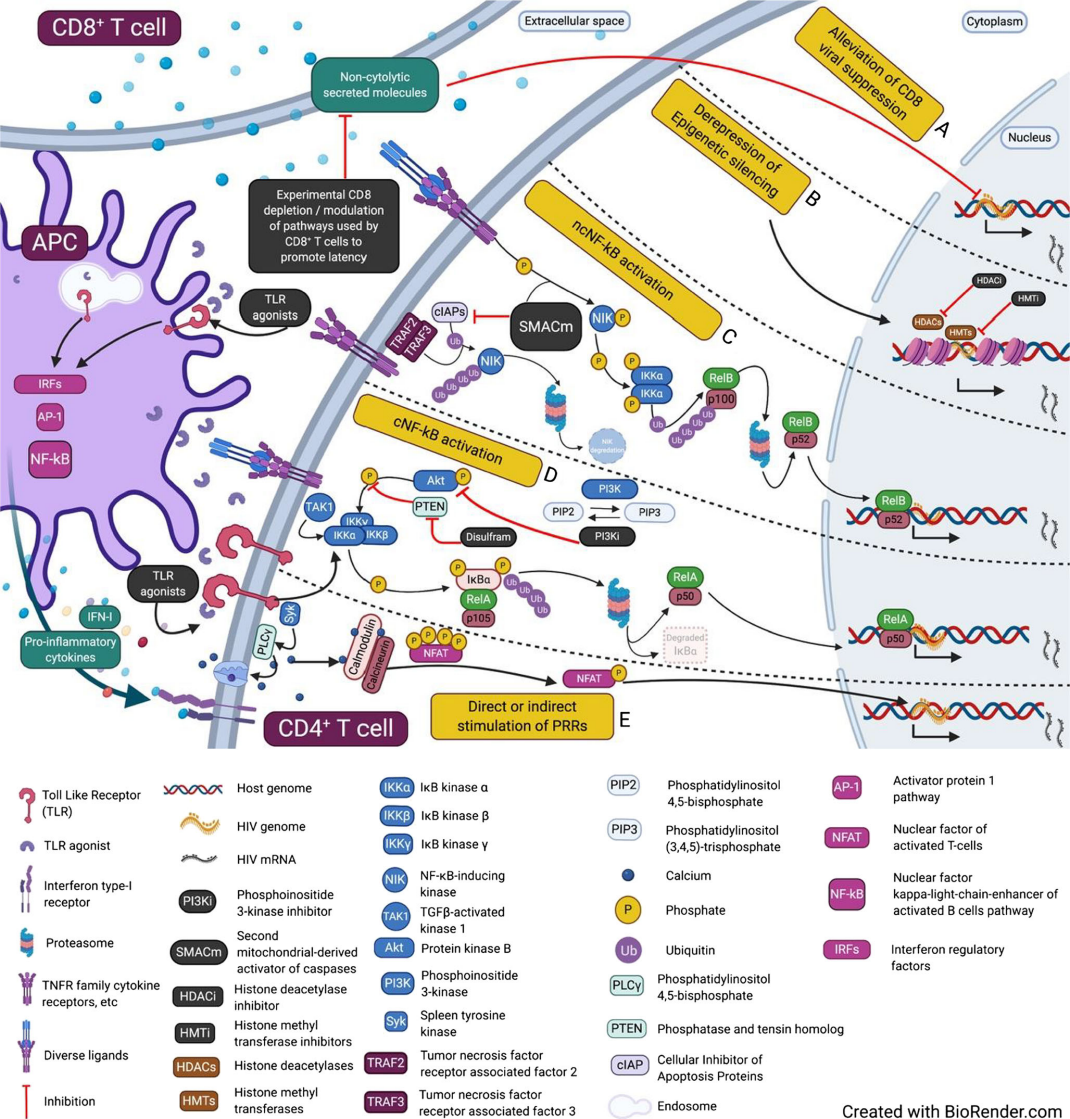
Latency reversal agents and their known or proposed mechanisms of action. (A) Alleviation of CD8 viral suppression: non-cytolytic molecules secreted by CD8+ T cells suppress HIV RNA production by mechanisms yet to be fully elucidated. Experimental CD8 depletion alleviates CD8+ T cell suppression leading to viral reactivation. Identifying the specific molecular pathways used by CD8+ T cells to promote latency might be a key to maximal latency reversal. (B) Derepression of epigenetic silencing: HDACi’s and HMTi’s unwind the genome thereby increasing access of transcription factors to targeted genes for expression. (C) Non-canonical NF-κB pathway activation: in a normal and unstimulated condition, NIK is regulated by the action of TRAF3 ligase binding to NIK and TRAF2 binding to cIAPs. This results in constant ubiquitination and degradation of NIK, ultimately maintaining low-levels of NIK. Under cellular stimulation by diverse ligands, receptor binding leads to TRAF3 degradation and consequent NIK accumulation, thereby enabling nuclear entry of the transcription factors of RelB and p52. This pathway can also be activated by binding of SMACm to cIAPs, resulting in NIK accumulation and similar downstream events. (D) Canonical NF-κB pathway activation: stimulation of a surface receptor leads to recruitment of TGFβ-activated kinase 1 (TAK1), activation of the IKK complex, and ultimately release of the transcription factors RelA and p50 to translocate to the nucleus. PI3K inhibitors prevent Akt activation thereby enabling nuclear entry of NF-κB. (E) Direct or indirect stimulation of PRR: stimulation of antigen presenting cells (APCs) via surface or endosomal TLR) activates the canonical NF-κB, AP-1, or IRF pathways resulting in secretion of pro-inflammatory cytokines (including type I interferons) which indirectly leads to latency reversal in CD4+ T cells. Stimulation of TLR on CD4+ T cells can also directly activate the canonical NF-κB or NFAT pathways. Similar activities of other PRR agonists have been described

New classes of molecules with immunomodulatory properties are being explored as potential LRA including pattern recognition receptor (PRR) ligands and immune checkpoint inhibitors (ICIs). These approaches have the theoretical advantage of simultaneously reactivating HIV and restoring immune functions to facilitate elimination of the infected cells. For the purpose of this review, we will focus on the latency reversal activity of these agents (Fig. 1).

### PRR Agonists

Pattern recognition receptors (PRR) are central players of innate immunity involved in pathogen sensing. Unlike agents that target epigenetic silencing, PRR agonists indirectly activate CD4^+^ T cells and HIV transcription presumably through stimulation of antigen presenting cells followed by pro-inflammatory cytokine release, although precise mechanisms have not been delineated for every agonist. Several toll-like receptor (TLR) agonists have reached pre-clinical and clinical testing of their ability to reverse HIV latency. The most extensively studied have been TLR-7 agonists that trigger type I interferon production by plasmacytoid dendritic cells (pDCs) with downstream activation of CD4+ T cells. In 2018, it was reported that two TLR-7 agonists (GS-986 and GS-9620) induced in vivo viral reactivation as well as reservoir reduction in the rhesus macaque (RM) model of simian immunodeficiency virus (SIV) infection and suppressive ART [29•]. Viral reactivation was demonstrated by transient increases in viremia while ART was maintained. Furthermore, 2/9 RM treated with GS-9620 did not show viral rebound after ART interruption and adoptive transfer of peripheral blood mononuclear cells (PBMCs) and lymph node mononuclear cells from these two RM to two uninfected animals did not result in establishment of infection. However, these exciting results showing virus reactivation were not reproduced in multiple additional studies in RM infected with SIV or simian human immunodeficiency virus (SHIV) [30•, 31•, 32•, 33, 34] nor in PLWH. Vesatolimod (formerly GS-9620) was assessed in a phase 1b, randomized, double-blind, placebo-controlled clinical trial, and only isolated viral load elevations above 20 copies/ml (highest 69 copies/ml) were observed [35].

The TLR-9 ligand MGN1703 that also stimulates type I interferon from pDCs was also advanced to clinical trials. A single-arm study reported quantifiable on-ART viremia (21–1571 copies/ml) in 6/15 HIV-1-infected individuals, during a short-course treatment with MGN1703 (also called lefitolimod) [36]. However, a subsequent study failed to demonstrate a virological benefit of MGN1703 administration with unchanged US CA HIV RNA levels and a stable reservoir as evaluated by total HIV DNA or replication-competent virus levels in CD4^+^ T cells and time to rebound following ATI [37]. An effect on on-ART viremia was not reported in this study and in a similar trial of a different TLR-9 agonist (CpG-ODN7909) [38], although presumably if significant increases were seen, these would have been included in the published results. The safety and efficacy of MGN1703 will be further assessed in combination with broadly neutralizing antibodies in a randomized clinical trial (NCT03837756). The TLR-3 agonist Poly-ICLC has also been evaluated but did not induce significant latency reversal [39]. Overall, the TLR agonists tested thus far have not proven to be reproducibly successful as LRA and most have the disadvantage of not directly acting upon CD4+ T cells.

In addition to the well-characterized TLR, cytosolic PRR have been explored as potential targets of the shock approach including retinoic acid-inducible gene-I (RIG-I)-like receptors (RLRs), and stimulator of interferon genes (STING). Acitretin, an FDA-approved retinoic acid derivative that enhances RIG-I signaling, has been shown to increase HIV transcription in vitro and induce preferential apoptosis of HIV-infected cells [40]. The STING pathway activates interferon regulatory factors and the nuclear factor kappa B (NF-kB) and could thus act indirectly through antigen presenting cells and directly on T cells. The STING ligands 2′3′-cGAMP and c-d-AMP have been reported to increase SIV RNA levels and decrease SIV DNA levels ex vivo in PBMCs isolated from cynomolgus macaques with natural control of viremia [41]. The combination of cGAMP and the HDACi resminostat was also shown to induce a significant increase in HIV reactivation and apoptosis in HIV-infected cells in vitro [42]. Ongoing work in our laboratory and that of M. Paiardini is exploring the virological and immunological impacts of STING agonist administration in vivo in RM during the acute phase of SIV infection or after sustained suppression of viremia on ART.

### Immune Checkpoint Inhibitors

The dysregulation of the immune system observed during chronic HIV infection involves a progressive exhaustion of CD8^+^ T cells characterized by the overexpression of co-inhibitory receptors, such as PD-1 (programmed cell death-1), CTLA-4 (cytotoxic T lymphocyte-associated protein 4), LAG-3 (lymphocyte-activation gene 3), TIGIT (T cell immunoreceptor with immunoglobulin and ITIM domains), or Tim-3 (T cell immunoglobulin and mucin domain-containing protein 3). In addition, co-inhibitory receptors are thought to contribute to HIV latency and are preferentially expressed at the surface of latently HIV-infected CD4+ T cells [43–48]. As such, blockade of co-inhibitory receptors represents a valuable therapeutic approach that could both restore immune functions of the exhausted HIV-specific T cells and reverse latency. As ICIs were initially developed for cancer therapy, several reports of their impact on HIV persistence come from observations of PLWH with coexisting malignancies. Increases in CA US HIV RNA in CD4+ T cells were observed following treatment with the anti-CTLA-4 antibody ipilimumab in an individual with metastatic melanoma, and interestingly, cyclic decreases in viremia using a single copy assay were also noted (rather than the increased on-ART viremia we would expect to see with effective latency reversal) [49]. This same individual received a single intravenous infusion of anti-PD-1 nivolumab resulting in a significant increase in CA US HIV RNA but again no significant change in plasma HIV RNA levels [44]. However, the impact of immune checkpoint inhibitors on latency reversal is inconsistent in these case reports [50–52]. Interestingly, a pre-clinical study in ART-suppressed SIV-infected RM treated with monoclonal antibodies targeting PD-1 and/or CTLA-4 suggested viral reactivation as demonstrated by on-ART viremia in a fraction of the treated animals as well as a reduction of the reservoir as shown by a significantly decreased level of cell-associated SIV DNA in effector memory CD4^+^ T cells and a decreased frequency of intact provirus [53••]. As autoimmune-related side effects have now been seen in multiple clinical trials of ICIs [54, 55], the future of this approach to reverse latency is uncertain.

## Non-canonical NF-κB Stimulation as LRA 2.0

### The NF-κB Pathway (Canonical vs Non-Canonical)

Recent work from our group and others has highlighted the promise of selective activation of the non-canonical NF-κB pathway for HIV and SIV latency reversal in CD4+ T cells. The NF-κB family includes 5 inducible transcription factors: NF-κB1 (p50), NF-κB2 (p52), RELA (p65), RELB, and c-REL. Activation of the classical or canonical NF-κB pathway, mediated by cell surface stimulation, recruitment of adaptor molecules to convert the IKK complex (inhibitor of nuclear factor κB), and a series of phosphorylation, ubiquitination, and finally degradation steps for IκB proteins, mostly triggers the transcription factors NF-κB1, RELA, and c-REL [56]. The canonical NF-κB pathway activates a diverse and broad range of genes and the response to stimuli is rapid and transient [57]. A second mode of NF-κB pathway activation, termed non-canonical, selectively and predominantly activates NF-κB2 and RELB in a strictly inducible manner through processing of p100. Response to stimuli in the non-canonical NF-κB (ncNF-κB) pathway is slow but persistent and transcription occurs in a more narrow set of genes compared to the canonical NF-κB pathway [57]. A central component of the ncNF-κB pathway is NIK (NF-κB-inducing kinase) through which all pathway inducers are known to signal [58]. In the unstimulated condition, newly synthesized NIK is constantly ubiquitinated and degraded by TRAF3 [59]. Specifically, TRAF3 recruits TRAF2 that binds to cellular inhibitor of apoptosis 1 and 2 (cIAP1, cIAP2). Cell surface binding of ligands to TNFRs (BAFFR, CD40, CD30, CD27, etc.) stimulates TRAF3 degradation, NIK accumulation, IKKa activation, and p100 phosphorylation and degradation. In addition to receptor ligation, the ncNF-κB pathway can be activated by intermediates of the apoptosis cascade such as the second mitochondrial activator of caspases (SMAC) that induce the degradation of cIAP and thus NIK activation. Peptide mimetics with SMAC-like activity, termed SMAC mimetics (SMACm), were developed initially to promote apoptosis in tumor cells and have now been shown to induce HIV and SIV latency reversal.

Protein kinase C agonists (PKCa) that activate NF-κB through the canonical pathway have been explored as LRA. However, due to toxicity concerns related to the potent and broad activation of signaling pathways, only one clinical trial has been performed to date. Administration of two different single doses of bryostatin-1 failed to induce HIV-1 RNA transcription as evaluated by US CA RNA levels in PBMCs and on-ART viremia in a pilot double-blind phase I clinical trial [60]. An alternative to PKCa is represented by disulfiram, a drug used to treat chronic alcoholism, that activates the phosphoinositide-3-kinase (PI3K)/Akt pathway that interacts with the NF-κB cascade resulting in nuclear entry of NF-κB1 and RELA (canonical transcription factors). Two clinical trials of short-term administration of disulfiram showed no to limited effect on HIV transcription with only a twofold increase in CA US HIV-1 RNA at the highest dose tested [61, 62]. In both studies, the viral reservoir size was not reduced following treatment with disulfiram.

### SMAC Mimetics

In 2015, Pache and colleagues showed that several SMACm induced increased levels of HIV transcription in latently infected Jurkat cells by stimulating the ncNF-κB pathway [63••]. A synergistic effect on latency reversal activity was also seen when combining SMACm with panobinostat in vitro. Similar results were observed with the SMACm Debio 1143 in a subsequent study showing latency reversal in vitro and ex vivo in resting CD4^+^ T cells isolated from ART-suppressed PLWH and humanized bone marrow/liver/thymic (BLT) mice [64].

Our group recently demonstrated that activation of the ncNF-κB signaling pathway by the SMACm AZD5582 reversed both HIV and SIV latency in vivo as shown by the induction of viral RNA expression in the blood and tissues of ART-suppressed HIV-infected BLT humanized mice and SIV-infected RM. ART-suppressed HIV-1JR-CSF-infected BLT mice were given a single injection of SMACm leading to on-ART viremia in > 50% of mice. Additionally, comparison of HIV RNA level in resting CD4^+^ T cells isolated from various tissues of ART-suppressed BLT mice including the bone marrow, thymic organoid, lymph node, spleen, liver, and lungs showed increased levels in SMACm-treated animals vs controls. Furthermore, we also evaluated AZD5582 latency reversal activity in twelve RM infected by SIV_mac239_ and treated with a potent ART regimen for over a year before receiving weekly infusions of SMACm for 3 or 10 weeks while ART was maintained. Increased expression of several mediators of the ncNF-κB pathway was confirmed by RNA sequencing, including NF-κB2 and RELB. On-ART viremia was observed in RM treated with AZD5582 (5/12 RM using a standard viral load assay and 8/12 RM using an ultrasensitive assay). The highest measurement was ~ 10^3^ copies/ml with multiple instances of sustained viremia between AZD5582 infusions. In the RM who received ten doses of AZD5582, cell-associated SIV RNA was also significantly higher in resting CD4^+^ T cells isolated from lymph nodes as compared to controls and the replication-competent reservoir in lymph node CD4+ T cells was reduced. No further impact of AZD5582 treatment on the viral reservoir size was observed [65••]. In unpublished work, we have found that treatment of SIV-infected ART-suppressed RM with a second cycle of the SMACm AZD5582 can induce on-ART viremia > 60 copies/ml in 75% of RM that were initially not responsive. More recently, Pache et al. replicated the finding of latency reversal in vivo using a humanized mouse model with the SMACm Ciapavir [66].

Although a robust decline in the CD4+ T cell reservoir was not observed in these studies, SMACm have been suggested to selectively target and kill HIV-infected resting memory CD4+ T cells and macrophages by an autophagy-dependent mechanism [67, 68]. Besides the potential for targeting apoptosis of infected cells, SMACm present the major advantage of being more specific than most LRA developed so far, thus limiting safety concerns. Collectively, these results suggest that activating the ncNF-κB pathway using SMACm is an efficient strategy to reactivate HIV/SIV that warrants further investigation.

## Transient CD8+ Lymphocyte Depletion as LRA 2.0

While it is known that CD8+ T cells utilize cytolytic mechanisms of viral control during HIV infection, a non-cytolytic role for CD8+ T cells in promoting viral latency has also been described. Several in vitro and in vivo studies have demonstrated that, both in the presence and absence of ART, CD8+ T cells exert a suppressive effect on virus production in HIV/SIV-infected CD4+ T cells. In early evidence from in vitro experiments, removal of CD8+ T cells from HIV-infected PBMCs consistently yielded high levels of HIV reverse transcriptase (RT) activity in supernatant in contrast with undepleted PBMC cultures from the same donors that was unrelated to an effect on cell killing [69]. A dose-dependent relationship between level of viral suppression (measured by reverse transcriptase activity) and number of CD8+ T cells with controlled reintroduction of autologous CD8+ T cells was found. A substantial portion of the more recent evidence for CD8+ cells promoting viral latency comes from studies in nonhuman primates in which experimental depletion of CD8+ lymphocytes can be accomplished by monoclonal antibody administration. The two commonly used antibodies for this purpose are MT-807R1 which targets the CD8α chain and is highly effective in transiently depleting CD8+ T and NK cells, and CD8b255R1 which targets the CD8β chain and selectively removes CD8+ T cells, albeit to a lesser extent and duration than MT-807R1 [70]. Below, we summarize the experimental data supporting this non-cytolytic role and the utility of such findings for latency reversal agents in curative research.

Studies of untreated SIV infection in which CD8a depletion was performed with MT-807R1 have demonstrated the efficacy of CD8+ T cells in controlling viral replication, and this research was extended to incorporate continuous ART administration after SIV/SHIV infection to model the latent state in vivo. In a cohort of SIV-infected RM, it was found that CD8+ T cells were required for maintaining viral suppression during short-term ART [71]. In 100% of animals undergoing CD8+ lymphocyte depletion with MT-807R1, plasma viral load increases were detected during the depletion period (> 90% depletion of circulating CD8+ T cells), and subsequent CD8+ T cell reconstitution correlated with reversion to viral control.

Following this initial paradigm shifting experiment, three independent in vivo studies from our group and that of G. Silvestri demonstrated enhanced latency reversal when CD8+ lymphocyte depletion was combined with another agent. Given substantial heterogeneity within the latently infected pool, combination LRA treatments are hypothesized to achieve more potent and broader virus reactivation. In the first study, a single administration of MT-807R1 was given prior to four weekly doses of the IL-15 superagonist (N-803) in SIV-infected, long-term ART-suppressed macaques [72••]. N-803 was selected based upon its latency reversal activity in vitro [73]. All macaques showed virus production in plasma post-CD8+ lymphocyte depletion and N-803 administration, some to levels as high as 10^4^ copies/ml, and on-ART viremia continued for several weeks until CD8+ T cells were reconstituted [72••]. Findings were replicated in HIV-infected humanized mice and in an in vitro latency and reversal assay (LARA) that uses autologous CD8+ T cells isolated prior to HIV infection, indicating that the suppressive effect of CD8+ T cells is not restricted to antigen-experienced, virus-specific T cells. In each of these experiments (SIV-infected macaques, HIV-infected humanized mice, and HIV-infected human PBMCs), the combination of CD8 depletion and N-803 showed significantly greater virus reactivation compared to CD8 depletion or N-803 administration alone; however, viral rebound dynamics were not altered in comparison to controls when ART was interrupted. As a proof-of-concept, we also demonstrated that suboptimal depletion of CD8+ T cells using the CD8b255R1 antibody given with four weekly doses of N-803 in ART-suppressed, SHIV-infected macaques induced virus reactivation in three out of five macaques [70]. Finally, the CD8a-depleting antibody was given in tandem with the SMACm AZD5582 that we have shown to be a highly effective LRA when used alone. The combination of CD8+ lymphocyte depletion and five weekly doses of the SMACm AZD5582 in SIV-infected ART-suppressed macaques resulted in on-ART viremia in 100% of treated animals [74]. As described earlier, in a similar cohort of ART-suppressed SIV-infected macaques, the effect of AZD5582 treatment alone was less extensive, with only 56% of animals experiencing viremia > 60 copies/ml [65••].

As it may not be practical to implement CD8 depletion studies in HIV-infected individuals, nailing down the precise mechanism(s) through which these cells are repressing virus production may aid in the identification of more targeted therapies that block this effect while CD8+ T cells remain.

A recent in vitro study aimed to better characterize the nature of the observed CD8+ T cell effect on latency and identified a non-MHC-dependent, non-cytolytic capacity of CD8+ T cells to suppress HIV replication through silencing of LTR-dependent viral transcription [75]. Co-culture of CD4+ T cells infected in vitro with the controlled addition of autologous (non-HIV exposed) CD8+ T cells resulted in increased levels of integrated HIV DNA, reduced CD4+ T cell activation, increased CD4+ T cell survival, and promotion of CD4+ T cell differentiation towards a Th2 profile. Together, these observations suggest that CD8+ T cells may favor the survival of resting CD4+ T cells carrying integrated HIV provirus.

## Promising LRA and LRA Combinations

In addition to the LRA mentioned above, there are several new agents or combinations of agents that we are likely to hear more about in the coming years, based on promising in vitro work. These include additional TLR stimulators, including a dual TLR-2 and -7 agonist [76], and others targeting TLR-8 [77], or TLR-1/2 that may act directly on CD4+ T cells [78]. Fimepinostat, that inhibits both PI3K and HDAC, was shown to increase levels of US HIV-1 RNA in CD4+ T cells from donors on long-term ART without causing activation of central or effector memory CD4+ T cells [79]. In another approach to specifically target intracellular signaling pathways in CD4+ T cells, Bosque and colleagues identified benzotriazoles as inhibitors of the SUMOlyation (and thus inactivation) of STAT5 [80]. Treatment of CD4+ T cells from ART-suppressed donors with benzotriazole + IL-2 resulted in p24 release without an increase in cellular activation or proliferation. Interestingly, the effect required IL-2, a member of the γ-chain cytokine family that also includes IL-15 and IL-7 which also activate STAT5 [81]. Several trials of IL-15 (as the superagonist N-803) alone or in combination with other agents are planned or underway [82]. IL-7 also has also been shown to induce p24 expression from thymocytes and splenocytes from HIV-1 infected SCID-hu mice stimulated ex vivo in the presence of ART [83]. As discussed above, IL-2 was toxic when given to HIV-infected trial participants, and while the in vitro results described in this section are encouraging, all of these approaches will need to be validated for safety and efficacy in pre-clinical nonhuman primate models or clinical trials.

Relatively new pharmacologic agents termed senotherapeutics function to target cells in their senescence, a state characterized by pro-inflammatory cell cycle arrest. Such compounds include senolytics which lead to elimination of senescent cells and senomorphics which inhibit or suppress the senescence-associated secretory phenotype (SASP) (that encompasses the collective secreted factors promoting inflammation during senescence) [84]. It has been previously shown that persistent immune activation due to chronic HIV infection may hasten immunosenescence, or immune aging [85]. This led to the investigation of select senotherapeutic compounds as potential HIV curative agents targeting latently infected CD4+ T cells through mechanisms including latency reversal, targeted apoptosis, and anti-proliferation [86]. The senomorphic mechanistic target of rapamycin (mTOR) inhibitor sirolimus (or rapamycin) has been studied for its anti-proliferative properties in latent HIV infection. With regard to latency reversal, Martin and colleagues showed that resting CD4+ T cells from ART-suppressed individuals showed equivalent induction of HIV mRNA upon αCD3/αCD28 stimulation in the presence or absence of rapamycin and, importantly, rapamycin was associated with significantly reduced production of pro-inflammatory cytokines [87]. In the same study, coupling of rapamycin with the PKC agonist bryostatin-1 or HDACi romidepsin did not significantly affect HIV mRNA production but did inhibit stimulation-induced cytokine release. A similar interaction has been reported between the PKC agonist ingenol B and the Janus kinase (JAK) inhibitor ruxolitinb [88]. Overall, this suggests that use of global T cell activators in the presence of mTOR or JAK inhibitors may reduce toxicity and ultimately open particular LRA 1.0 candidates for reconsideration.

## LRA 2.0 in Shock and Kill Strategies

A stable viral reservoir size following LRA administration suggests an absence of or insufficient clearance of infected cells by virus-induced cytopathic effect or unboosted immune effector cells. With better understanding of latency reversal and now several approaches available that induce readily measurable on-ART viremia, several groups are thus exploring in vivo combination LRA 2.0 plus clearance agents in nonhuman primate models. We recently used bispecific HIVxCD3 retargeting molecules in combination with the SMACm AZD5582 in a model of ART-suppressed SHIV-infected RM [89], but unexpectedly did not observe latency reversal that we attributed to a small reservoir size in this model. We are currently using the model of ART-suppressed RM infected with SIV to assess the impact on the viral reservoir of a combination of a cocktail of SIV-specific neutralizing and non-neutralizing monoclonal antibodies given with either AZD5582 or N-803 plus the CD8α-depleting antibody MT-807R1. Additional approaches that may prove successful include “shock and suicide” in which reactivated cells are specifically induced to die with inhibitors of prosurvival proteins or “surge and purge” in which both the LRA/immune stimulator and clearance agent are given coincident with ART initiation early in infection to restrict reservoir establishment.

## Remaining Questions About Latency Reversal as a Component of an HIV Cure Strategy

With the recent successes in achieving robust latency reversal in multiple animal models as described above, our attention is now focused on capitalizing on these LRA in combination shock and kill experiments that include a clearance arm to reduce or eliminate persistent reservoirs. However, several outstanding questions remain regarding the effect and consequences of latency reversal with SMACm and/or N803 + CD8 lymphocyte depletion. First, what are the specific cellular and anatomic origins of reactivated virus that lead to on-ART viremia; second, might these immune stimulating approaches have the unintended consequence of expanding the reservoir through promotion of cellular proliferation and clonal expansion; third, what depth and extent of latency reversal is needed to provide sufficient fodder for clearance agents to measurably change the reservoir size; and fourth, is there a differential susceptibility of CD4+ T cell subsets with reactivated virus to diverse elimination strategies? The answer to these questions will come from future studies that not only assess the efficacy of LRA to reactivate latent virus but also specifically address these more mechanistic considerations. Prioritizing experiments designed to dig deeper into the consequences of latency reversal and using the information gained to optimize approaches to eliminate reactivated cells is likely to significantly advance the HIV cure field.
